# Modeling Niemann-Pick disease type C1 in zebrafish: a robust platform for *in vivo* screening of candidate therapeutic compounds

**DOI:** 10.1242/dmm.034165

**Published:** 2018-08-15

**Authors:** Wei-Chia Tseng, Hannah E. Loeb, Wuhong Pei, Chon-Hwa Tsai-Morris, Lisha Xu, Celine V. Cluzeau, Christopher A. Wassif, Benjamin Feldman, Shawn M. Burgess, William J. Pavan, Forbes D. Porter

**Affiliations:** 1 Section on Molecular Dysmorphology, Division of Translational Research, Eunice Kennedy Shriver National Institute of Child Health and Human Development, National Institutes of Health, Department of Health and Human Services, Bethesda, MD 20892, USA; 2 Translational and Functional Genomics Branch, National Human Genome Research Institute, National Institutes of Health, Department of Health and Human Services, Bethesda, MD 20892, USA; 3 Zebrafish Core, Eunice Kennedy Shriver National Institute of Child Health and Human Development, National Institutes of Health, Department of Health and Human Services, Bethesda, MD 20892, USA; 4 Genetic Disease Research Branch, National Human Genome Research Institute, National Institutes of Health, Department of Health and Human Services, Bethesda, MD 20892, USA

**Keywords:** Drug screening, Liver disease, Lysosomal storage disorder, Neurodegeneration, Niemann-Pick disease type C1, Zebrafish

## Abstract

Niemann-Pick disease type C1 (NPC1) is a rare autosomal recessive lysosomal storage disease primarily caused by mutations in *NPC1*. NPC1 is characterized by abnormal accumulation of unesterified cholesterol and glycolipids in late endosomes and lysosomes. Common signs include neonatal jaundice, hepatosplenomegaly, cerebellar ataxia, seizures and cognitive decline. Both mouse and feline models of NPC1 mimic the disease progression in humans and have been used in preclinical studies of 2-hydroxypropyl-β-cyclodextrin (2HPβCD; VTS-270), a drug that appeared to slow neurological progression in a Phase 1/2 clinical trial. However, there remains a need to identify additional therapeutic agents. High-throughput drug screens have been useful in identifying potential therapeutic compounds; however, current preclinical testing is time and labor intensive. Thus, development of a high-capacity *in vivo* platform suitable for screening candidate drugs/compounds would be valuable for compound optimization and prioritizing subsequent *in vivo* testing. Here, we generated and characterize two zebrafish *npc1*-null mutants using CRISPR/Cas9-mediated gene targeting. The *npc1* mutants model both the early liver and later neurological disease phenotypes of NPC1. LysoTracker staining of *npc1* mutant larvae was notable for intense staining of lateral line neuromasts, thus providing a robust *in vivo* screen for lysosomal storage. As a proof of principle, we were able to show that treatment of the *npc1* mutant larvae with 2HPβCD significantly reduced neuromast LysoTracker staining. These data demonstrate the potential value of using this zebrafish NPC1 model for efficient and rapid *in vivo* optimization and screening of potential therapeutic compounds.

This article has an associated First Person interview with the first author of the paper.

## Abstract (precis)

**Summary:** A zebrafish genetic model of Niemann-Pick disease type C1 is suitable for performing *in vivo* screening of candidate therapeutic compounds by examining LysoTracker staining intensity in neuromasts.

## INTRODUCTION

Niemann-Pick disease type C (NPC) is a rare autosomal recessive disease caused by the accumulation of cholesterol and glycolipids in late endosomes/lysosomes. It is estimated to affect 1 in 90,000 individuals (Vanier, 2010; Wassif et al., 2016). NPC patients manifest a broad and heterogeneous range of symptoms/signs, including neonatal jaundice, hepatosplenomegaly, ataxia, tremor, seizures and learning difficulties (Patterson et al., 2013; Vanier, 2010). Infants frequently present with liver disease that can range from prolonged cholestatic jaundice to severe cirrhosis and liver failure. The onset of neurological manifestations of NPC is variable and ranges from early-infantile to adolescent/adult onset (Mengel et al., 2017). Mutation of both alleles of either *NPC1* or *NPC2* can cause NPC. Mutations in *NPC1* are reported in 95% of NPC patients, with mutations of *NPC2* accounting for the remaining cases (Vanier, 2010). Both NPC1 and NPC2 are lysosomal proteins that facilitate intracellular cholesterol trafficking (Carstea et al., 1997; Naureckiene et al., 2000; Vanier and Millat, 2004).

Intracellular cholesterol trafficking starts with low density lipoprotein (LDL) entering cells via LDL-receptor-mediated endocytosis. LDL particles are then disassembled in late endosomes/lysosomes, releasing cholesterol esters (Brown and Goldstein, 1986). Lysosomal acid lipase metabolizes cholesterol esters to yield unesterified cholesterol. NPC2 is a soluble, luminal, lysosomal protein that can bind cholesterol and transfer it to the N-terminal domain of NPC1 (Cruz et al., 2000; Kwon et al., 2009). NPC1 is a transmembrane glycoprotein located in the membrane of late endosomes/lysosomes (Ioannou, 2000). NPC1 in concert with NPC2 facilitates transport of unesterified cholesterol from the endolysosomal lumen to other cellular compartments such as the endoplasmic reticulum (Cruz et al., 2000; Gong et al., 2016; Li et al., 2016). This intracellular cholesterol transport is blocked in cells lacking functional NPC1 or NPC2, resulting in the accumulation of unesterified cholesterol and other lipids in late endosomes/lysosomes (Liscum et al., 1989; Wojtanik and Liscum, 2003). *NPC1* is highly conserved among many species, and both murine and feline NPC1 models manifest pathological and clinical findings similar to those observed in NPC1 patients (Higaki et al., 2004; Loftus et al., 1997; Maue et al., 2012; Miyawaki et al., 1982; Munana et al., 1994). Neurodegeneration, particularly a loss of cerebellar Purkinje neurons, is a major manifestation of NPC1 in both humans and animal models (Sarna et al., 2003; Vanier, 2010). These animal models have proven to be useful both in studying pathological processes and in preclinical testing of potential therapies. Specifically, both the mouse (Aqul et al., 2011; Davidson et al., 2009; Liu et al., 2009; Ramirez et al., 2010) and feline (Vite et al., 2015) models have proven invaluable for preclinical studies related to the development of 2-hydroxypropyl-β-cyclodextrin (2HPβCD). Although intrathecal VTS-270, a specific 2HPβCD, was shown to be effective in slowing neurological disease progression in NPC1 patients (Ory et al., 2017), additional therapeutic agents that either complement VTS-270 or alleviate the need for intrathecal administration need to be developed. High-throughput drug screens using NPC1-deficient cell lines have been conducted and have identified a number of potential therapeutic compounds (Rosenbaum et al., 2009; Rujoi et al., 2010; Yu et al., 2014). These *in vitro* findings need to be tested *in vivo*; however, *in vivo* testing in the current preclinical models is both time consuming and costly. Thus, an initial high-capacity *in vivo* screen of candidate drugs/compounds would be of significant utility to both optimize candidate compounds and to prioritize subsequent *in vivo* testing in mammalian models.

The development of CRISPR/Cas9-mediated gene targeting technology has greatly facilitated the use of zebrafish to model human genetic diseases (Hwang et al., 2013a,b). Zebrafish are a popular model organism for developmental biology studies due to the availability of genetic tools, large numbers of embryos produced from each cross and the transparency of the animal during embryonic development (Driever et al., 1994), which are also significant advantages for *in vivo* drug screening. Thus, to facilitate drug screening and optimization, we developed a genetic zebrafish NPC1 model utilizing CRISPR/Cas9-mediated gene targeting to mutate *npc1*. The *npc1*-null zebrafish manifests both liver and nervous system pathology, thus providing a model for both the peripheral and central nervous system (CNS) defects found in NPC1 patients. Furthermore, we demonstrate the utility of this novel model system as a rapid, high-capacity, *in vivo* screen of candidate therapeutic drugs.

## RESULTS

### Generation of *npc1*-null mutant zebrafish

**Fig. 1.**
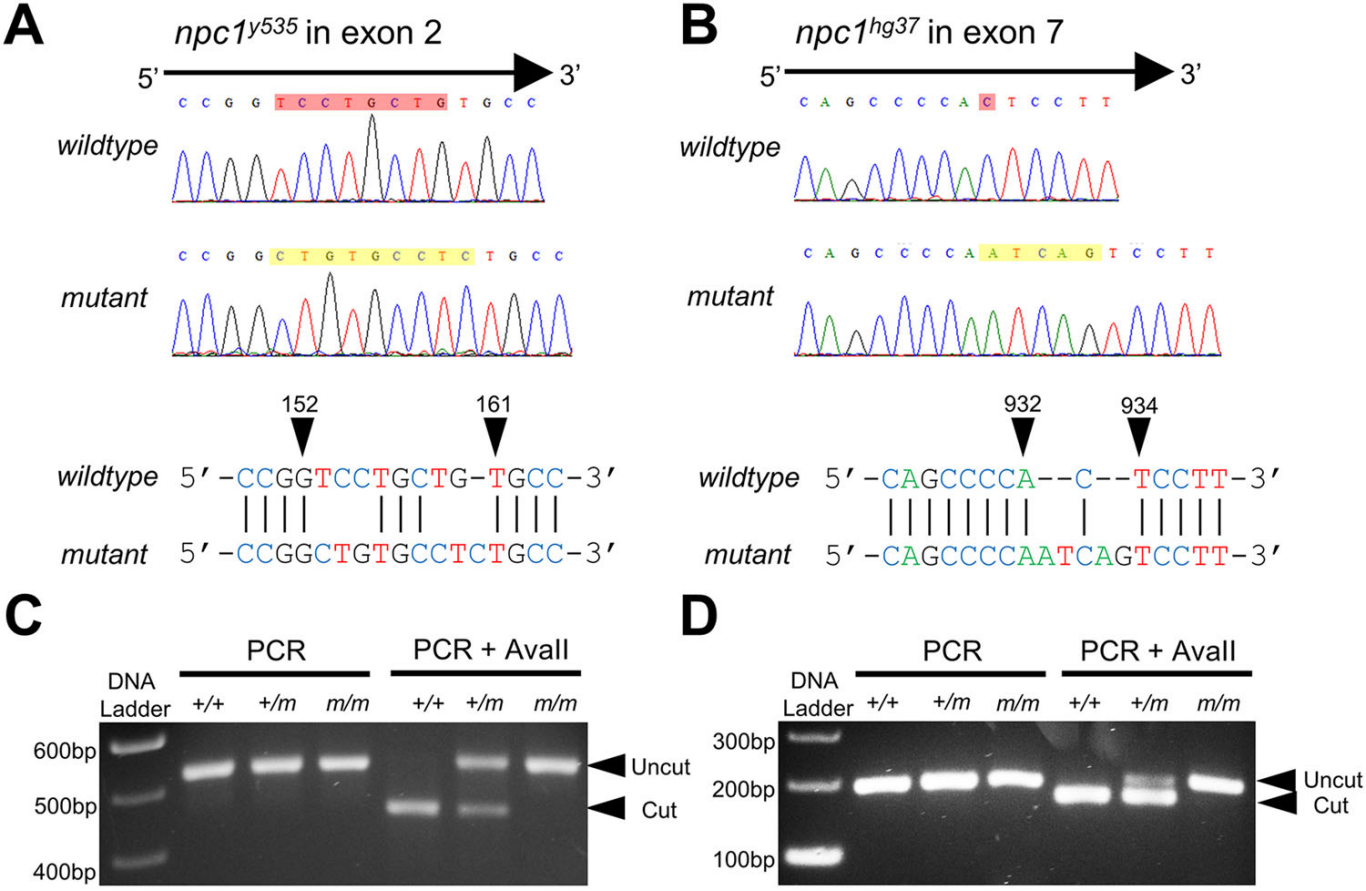
**Two zebrafish *npc1* mutant alleles were generated using CRISPR/Cas9-mediated gene targeting.** (A,B) Sequencing chromatographs of *npc1^y535^* (A), *npc1^hg37^* (B) and wild-type alleles. Arrowheads delineate the area of the induced insertion/deletion and indicate nucleotide positions corresponding to the wild-type *npc1* gene sequence. (C) The *npc1^y535^* allele disrupts an endogenous *Ava*II restriction site and genotyping can be performed by restriction fragment length polymorphism analysis. (D) Derived cleaved amplified polymorphic sequence (dCAPS) was used to introduce an *Ava*II restriction site into PCR products derived from the *npc1^hg37^* allele that was used for genotyping.

Zebrafish have a single *npc1* gene (NCBI Gene ID: 553330), which maps to chromosome 2. The zebrafish *npc1* gene consists of 25 coding exons encoding Npc1, a 1276 amino acid, lysosomal transmembrane protein. To generate *npc1*-null mutants, CRISPR/Cas9-mediated gene targeting was used to induce double-strand DNA breaks and error-prone repair in wild-type zebrafish. Two independent sites located within exon 2 and exon 7 were selected as the single guide RNA (sgRNA) targeting sites to increase the chance of generating mutations that would disrupt the Npc1 protein near the N-terminus and give rise to a non-functional *npc1* allele. Wild-type zebrafish embryos (F0) were injected with *npc1*-specific sgRNA and *cas9* mRNA at the 1-cell stage to induce somatic and germline mutations. The resulting F0 zebrafish were raised to adulthood and outcrossed to individual wild-type adults to obtain F1 embryos. The F1 embryos were screened for germline transmission of *npc1* mutations by PCR and fragment analysis. F0 adults carrying potential mutations in the germline were selected and outcrossed to individual wild-type adults. F1 embryos obtained from this second outcross were raised to adulthood and individually screened for *npc1* mutations. Two *npc1* frameshift mutant alleles, *npc1^y535^* and *npc1^hg37^*, were identified. The *npc1^y535^* allele (Fig. 1A) consists of a deletion of 8 nucleotides and insertion of 9 nucleotides in exon 2 [NCBI Reference Sequence: NM_001243875.1 (npc1_v001): c.153_160delinsCTGTGCCTC]. This results in a frameshift of 1 nucleotide and the formation of a stop codon 6 amino acids after the mutation site. The *npc1^hg37^* allele (Fig. 1B) consists of a deletion of 1 nucleotide and insertion of 5 nucleotides in exon 7 [NM_001243875.1 (npc1_v001): c.933delinsATCAG]. This results in a frameshift of 4 nucleotides and a premature stop codon 6 amino acids downstream of the mutation site. An endogenous *Ava*II restriction enzyme site is disrupted in the *npc1^y535^* allele, providing a useful assay for genotyping (Fig. 1C). The *npc1^hg37^* allele neither destroys nor creates a restriction enzyme site. Therefore, a forward primer was designed to generate an *Ava*II restriction site in the wild-type allele, but not in the mutant allele for genotyping using a derived cleaved amplified polymorphic sequences assay (dCAPS assay). Genotyping for the *npc1^hg37^* allele using the dCAPS assay is shown in Fig. 1D. Given the early premature termination of the putative protein, both alleles are expected to be null alleles. This was confirmed by western blot for Npc1 protein (Fig. S1). Consistent with being null alleles, the mutant phenotype was similar for both *npc1^hg37^* and *npc1^y535^*. Subsequent data reported in this paper correspond to the *npc1^hg37^* allele.

### *npc1* mutant zebrafish manifest growth retardation and premature lethality

**Fig. 2.**
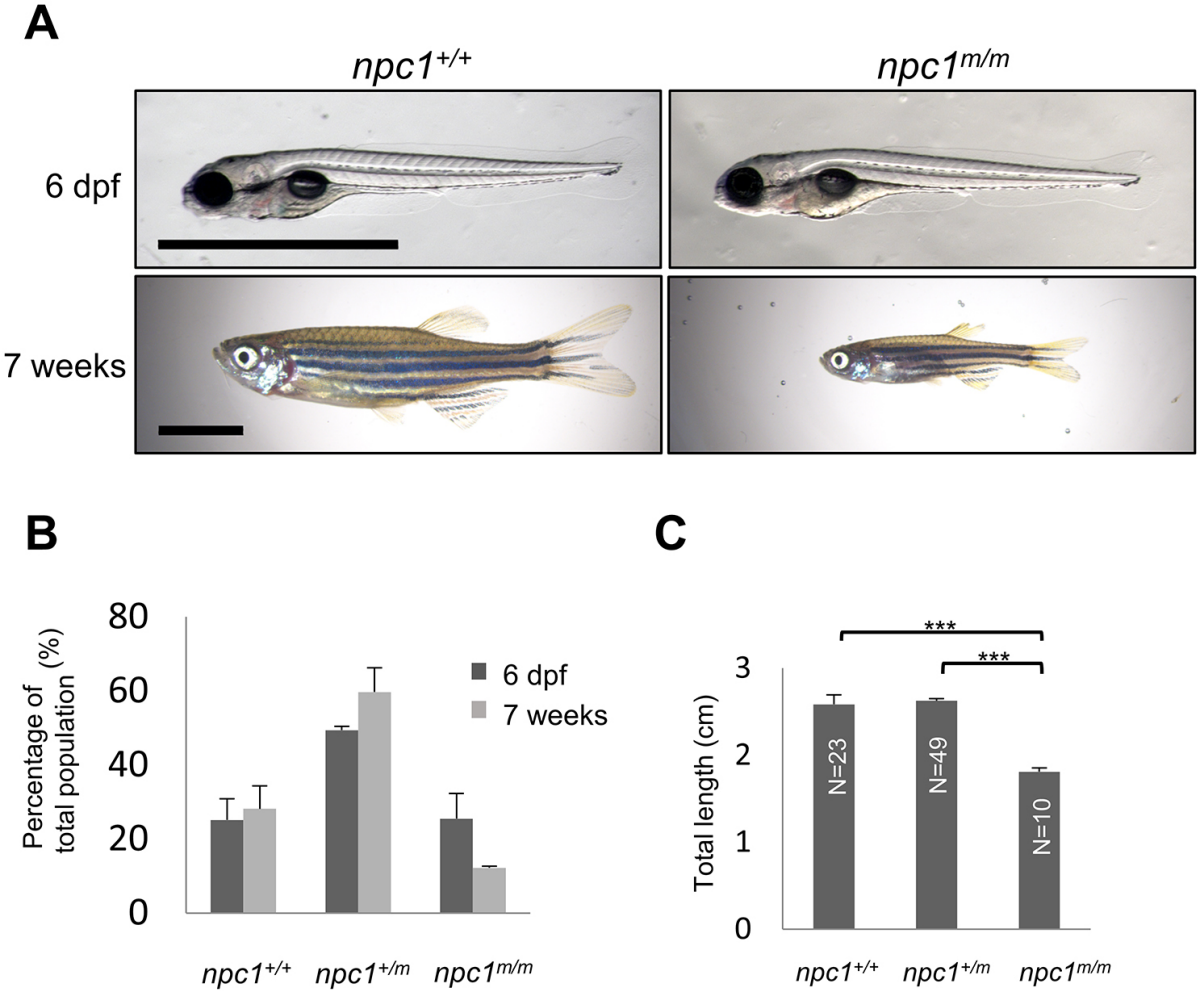
***npc1* mutant zebrafish morphology and survival.** (A) Photographs of representative wild-type and *npc1^m/m^* at 6 dpf and 7 weeks of age. No dysmorphology was noted at 6 dpf. Marked growth retardation was apparent at 7 weeks of age. Scale bars: 1 mm (6 dpf) and 5 mm (7 weeks). (B) Genotype distribution from the intercross of *npc1^+/m^* zebrafish at 6 dpf (*n*=166) and 7 weeks (*n*=82) of age. (C) Total length of *npc1^+/+^*, *npc1^+/m^* and *npc1^m/m^* zebrafish at 7 weeks of age. ***P<0.001 by two-tailed *t*-test.

**Fig. 3.**
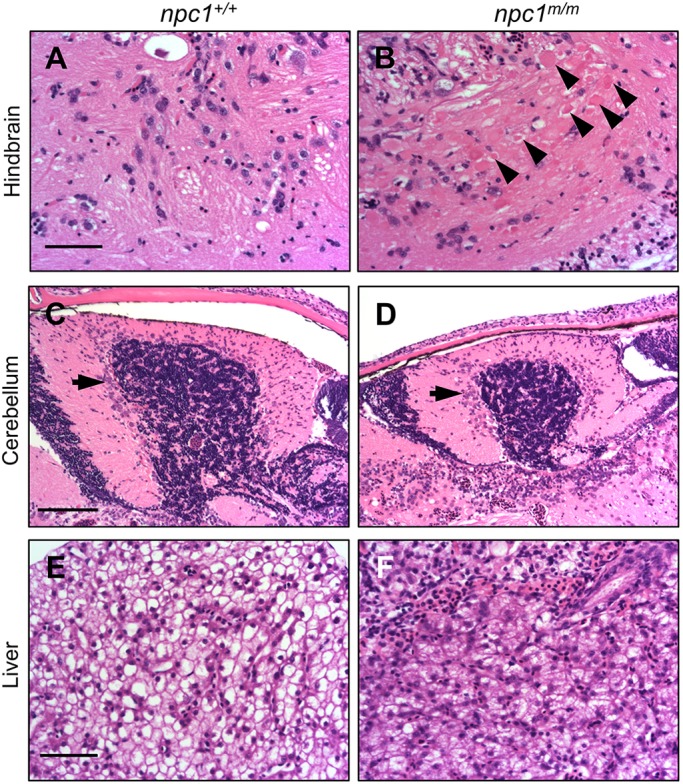
**Histopathology of adult *npc1^m/m^* liver and brain tissue.** (A,B) Photographs of Hematoxylin and Eosin (H&E)-stained hindbrain tissue sections from adult *npc1^+/+^* (A) and *npc1^m/m^* (B) zebrafish at 9 weeks of age. Axonal spheroids are indicated by the arrowheads. Scale bar: 50 μm. (C,D) H&E-stained cerebellar sections from 9-week *npc1^+/+^* (C) and *npc1^m/m^* (D) zebrafish. The arrows indicate cerebellar Purkinje neurons. Scale bar: 100 μm. (E,F) H&E-stained liver sections from 9-week *npc1^+/+^* (E) and *npc1^m/m^* (F) zebrafish. Scale bar: 50 μm.

NPC1 patients manifest a variety of visceral and neurological signs/symptoms at different ages. In severe NPC1 cases, lethal, prenatal onset has been described (Gumus et al., 2017), suggesting that disease progression might impact embryonic development. Furthermore, prior studies with targeted morpholino antisense oligonucleotides against *npc1* mRNA to knock down Npc1 protein expression in zebrafish showed defects in epiboly movement, notochord and somite development, as well as a defect in platelet formation (Louwette et al., 2013; Schwend et al., 2011). Therefore, we first examined whether there were any defects in zygotic *npc1^hg37^* mutants obtained from the intercross of *npc1^hg37^* heterozygous mutants. The three genotypes obtained from the intercross are referred to as *npc1^+/+^*, *npc1^+/m^* and *npc1^m/m^*, with ‘+’ and ‘m’ being used to designate the wild-type and mutant alleles, respectively. *npc1^m/m^* larvae appear to develop normally up to 6 days post-fertilization (dpf), with gross morphology essentially identical to their wild-type (*npc1^+/+^*) and heterozygous (*npc1^+/m^*) siblings (Fig. 2A). We also did not observe any increased death during this period. At 6 dpf, we observed the expected Mendelian distribution of genotypes, with 26.0±6.4% *npc1^m/m^* (*n*=166, Fig. 2B). In contrast, at 7 weeks of age (adolescent/adult stage), only 12.2±0.4% (*n*=82) of the surviving animals were homozygous *npc1* mutants (*P*<0.05, chi-square test). These data suggest significant mortality of *npc1^m/m^* zebrafish between late larval and adolescent stages. Early loss of *npc1^m/m^* occurred between 8 and 12 dpf. Mutants that were viable at 14 dpf typically survived until adult stages. Phenotypically, the surviving *npc1* mutants were significantly smaller than *npc1^+/+^* and *npc1^+/m^* siblings at 7 weeks of age (Fig. 2A,C). The surviving *npc1^m/m^* fish typically died prior to 6 months of age, whereas wild-type and heterozygous siblings typically live to more than 1 year of age. The adult *npc1* mutants were observed to develop a balance defect characterized by failure to maintain an upright position during swimming. Having developed this balance defect, adult *npc1* mutants exhibited a rapid spinning and tumbling movement and would subsequently die within a few days. The balance defect observed in the adult *npc1* mutants is likely due to a CNS defect. Histopathological studies were notable for axonal spheroids in the hindbrain of adult *npc1^m/m^* fish (Fig. 3A,B) and disorganized Purkinje neurons in the cerebellum (Fig. 3C,D). Specifically, the Purkinje neurons in the cerebellum of adult *npc1* mutants were more diffuse than the Purkinje neurons in control fish, which were found in a compact layer surrounding the granular layer. Axonal spheroids and cerebellar Purkinje cell loss are neuropathological findings described in NPC1 patients and mammalian models (Kodachi et al., 2017; Loftus et al., 1997; Maue et al., 2012; Miyawaki et al., 1982; Munana et al., 1994). Although some *npc1* mutants can survive to adulthood, they did not reproduce. Thus, we were limited to the evaluation of zygotic mutants in this study. The etiology of the growth retardation and apparent infertility are not known, and these phenotypes along with a more detailed neuropathological characterization will require further investigation. Although polymorphisms in the *NPC1* gene have been associated with obesity (Garver et al., 2015), impaired growth is not typically observed in NPC1 patients. The growth defect in the *npc1* mutant zebrafish may be due to impaired feeding, a common neurological problem found in NPC1 patients.

### Liver disease in *npc1* larvae

**Fig. 4.**
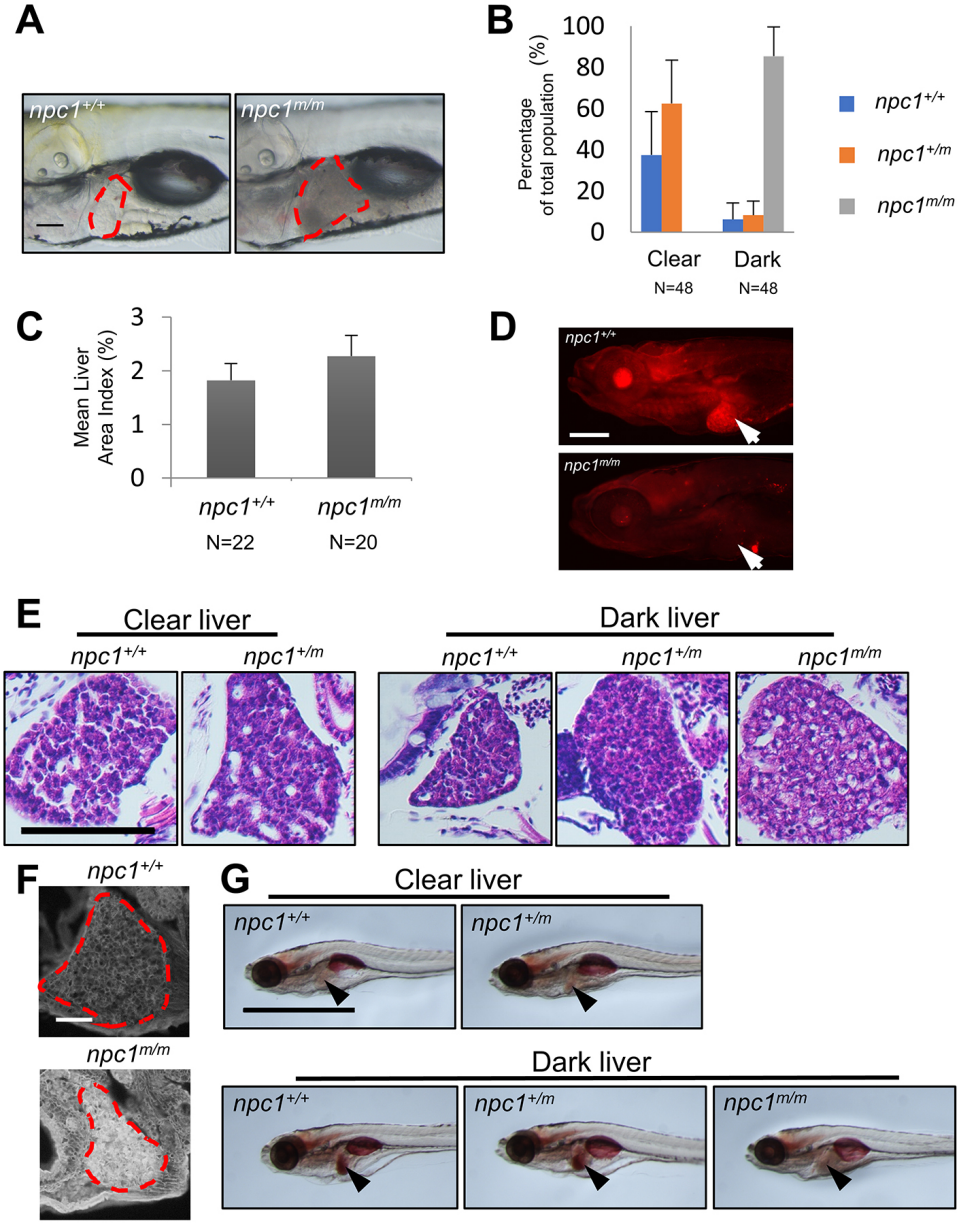
**Liver pathology in *npc1^m/m^* larvae.** (A) Lateral live images of *npc1^+/+^* and *npc1^m/m^* 7 dpf larvae. The area corresponding to liver is outlined by the red dashed line. Scale bar: 100 μm. (B) 7 dpf zebrafish larvae were separated by liver phenotype (clear versus dark) and then genotyped. A small proportion of *npc1^+/+^* or *npc1^+/m^* larvae had a dark liver phenotype. In contrast, all *npc1^m/m^* larvae had the dark liver phenotype. (C) Liver size was quantified by measuring the lateral view area corresponding to the liver tissue and normalized to the total lateral body projection area (area index) in 7 dpf larvae. Liver size was significantly (*P*<0.001, two-tailed *t*-test) increased in *npc1^m/m^* larvae compared to *npc1^+/+^* control larvae. (D) Immunofluorescence images of 7 dpf wild-type and *npc1^m/m^* stained with anti-NPC1 antibody. Arrows point to the liver. NPC1 staining is absent in the *npc1^m/m^* liver tissue. Scale bar: 200 μm. (E) H&E staining of liver tissue from 7 dpf *npc1^+/+^* and *npc1^+/−^* larvae with the clear liver phenotype, and *npc1^+/+^*, *npc1^+/m^* and *npc1^m/m^* larvae with the dark liver phenotype. Scale bar: 50 μm. (F) Filipin staining of 7 dpf *npc1^+/+^* and *npc1^m/m^* larvae. Liver tissue is outlined. Scale bar: 50 μm. (G) Oil Red O staining of 7 dpf larvae sorted with respect to clear/dark liver phenotype and genotype. Arrowheads point to the liver. Scale bar: 1 mm.

Liver disease can be a significant cause of morbidity and mortality in NPC1 infants (Kelly et al., 1993). In adult *npc1*^*m/m*^ fish, we observed that hepatocytes were full of cytoplasmic, vacuole-like structures (Fig. 3E,F). Given the significant mortality of *npc1^m/m^* between 6 dpf and 7 weeks, we evaluated the mutant larva for evidence of early liver disease. At 6 dpf, we observed no significant differences between control and mutant larva; however, at 7 dpf, a time point used to screen for post-developmental liver defects (Kim et al., 2011, 2015), we observed that approximately 25% of the larvae had dark and opaque livers, while the remaining larvae displayed clear and transparent livers (Fig. 4A). After genotyping it became apparent that the dark liver phenotype was predominately found in *npc1^m/m^* larvae (Fig. 4B). Although dark livers were occasionally observed in control larvae, a large majority of the larvae with the dark liver phenotype were *npc1* homozygous mutants (85.4±14.2%, *n*=48). Furthermore, none of the *npc1* mutant larvae had clear livers. Liver size was also significantly increased in the 7 dpf *npc1^m/m^* larvae (Fig. 4A,C). Immunofluorescence staining confirmed lack of Npc1 protein expression in *npc1^m/m^* livers at 7 dpf (Fig. 4D). Histopathological analysis from liver sections of 7 dpf larvae further showed that hepatocytes in *npc1* mutants with dark livers were larger and full of vacuole-like structures, whereas hepatocytes from larvae with a clear liver phenotype had a more compact and eosinophilic appearance (Fig. 4E). Hepatocytes from wild-type and heterozygous mutants with a dark liver phenotype were also more like those observed in larvae with the clear liver phenotype, although the organization was denser with fewer bile canaliculi (Fig. 4E). These histopathological differences suggested that the pathological mechanism giving rise to the dark liver phenotype in the *npc1^+/+^* and *npc1^+/m^* larvae was different from that occurring in the *npc1* mutant larvae. To further characterize the histopathology of the mutant dark liver phenotype, we stained the liver tissue with filipin, a fluorescent antibiotic that binds to unesterified cholesterol. Increased filipin staining is the histopathological hallmark of NPC disease. The intense filipin staining observed in liver tissue from 7 dpf *npc1^m/m^* larvae confirmed the accumulation of unesterified cholesterol (Fig. 4F). Dark-appearing, Oil Red O (ORO)-positive liver tissue in 7 dpf zebrafish larvae has been associated with steatosis (Kim et al., 2015). We thus evaluated ORO staining in this model. Liver tissue in *npc1* mutants were negative for ORO staining. In contrast, the wild-type or heterozygous siblings with a dark liver phenotype were positive for ORO staining. Thus, consistent with the different histopathological appearance, the ORO staining also suggests that the etiology of the dark liver phenotype differs between *npc1* mutants and their wild-type and heterozygous siblings (Fig. 4G).

### *npc1* mutants accumulate unesterified cholesterol in extraembryonic tissue

**Fig. 5.**
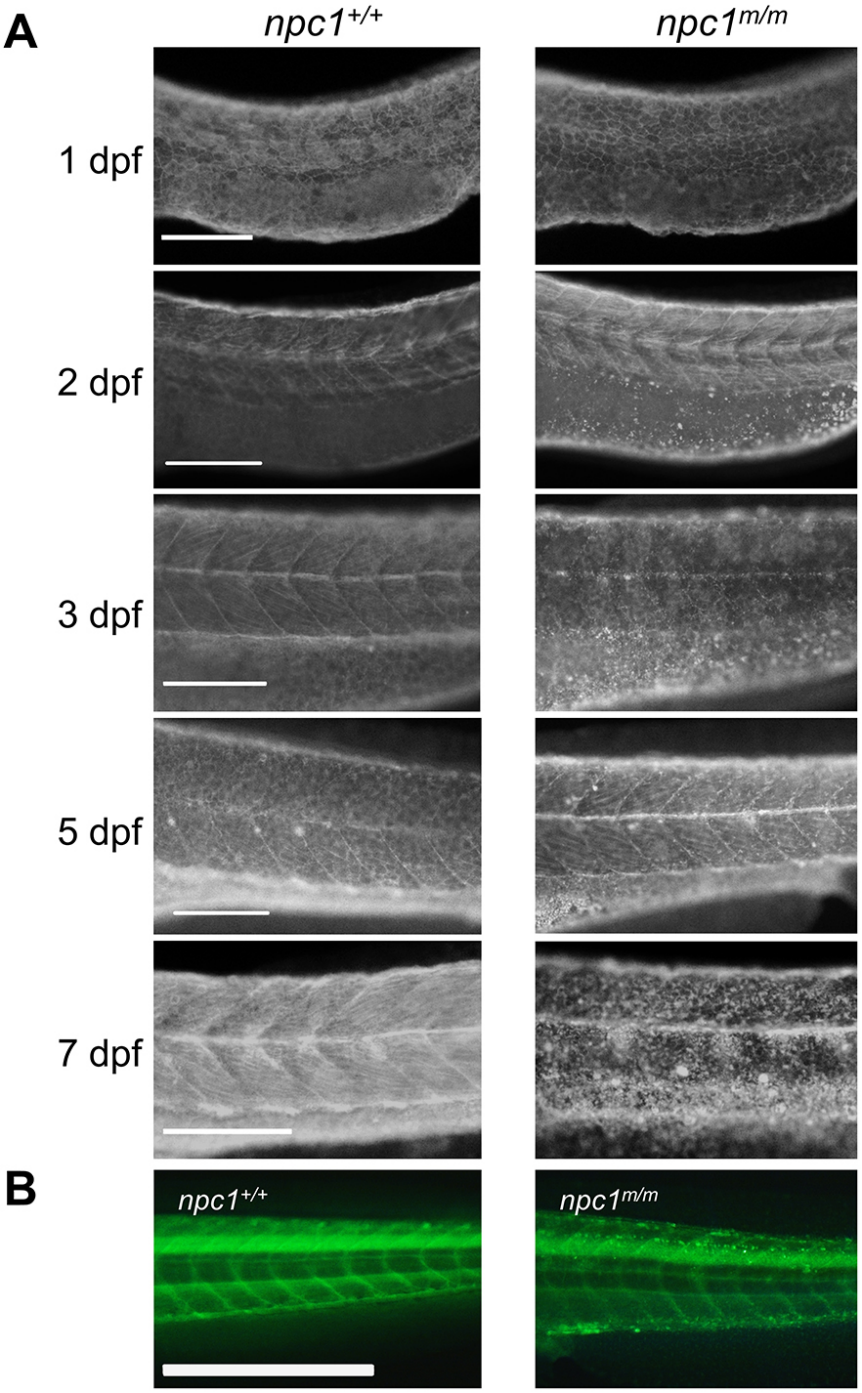
**Unesterified cholesterol accumulation.** (A) Control and mutant larvae were stained with filipin to visualize unesterified cholesterol. Filipin-positive puncta were initially observed in mutant larvae at 2 dpf in the area of the yolk sac extension and became more numerous with a wider distribution in older larvae. Scale bars: 200 µm. (B) Embryos were injected with TopFluor-cholesterol at the 1-cell stage and imaged at 7 dpf. Puncta of accumulated cholesterol were observed in the *npc1^m/m^* larvae. Scale bar: 1 mm.

**Fig. 6.**
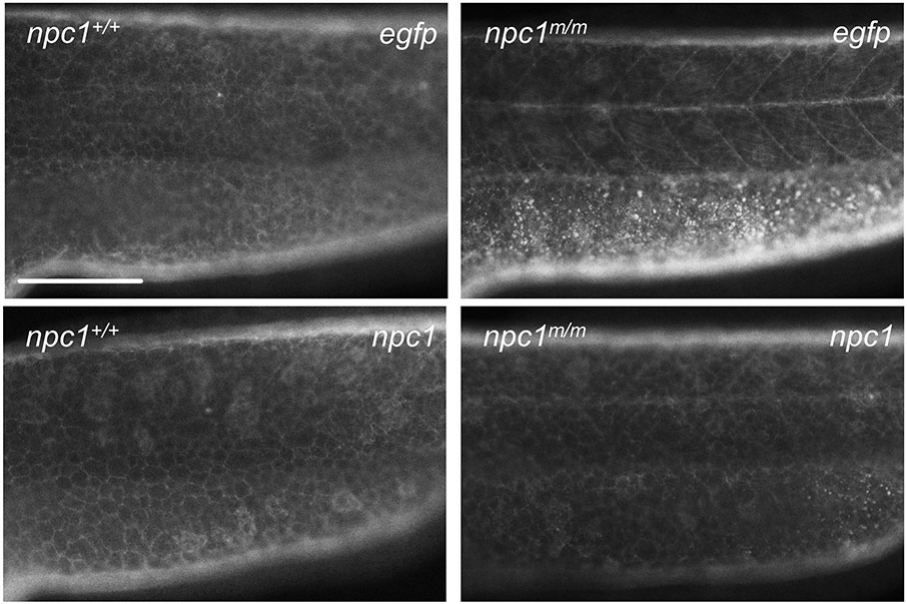
**Yolk syncytial layer accumulation of unesterified cholesterol.** Plasmids expressing either *EGFP* or *npc1* were injected into the yolk syncytial layer (YSL) of 3.5 hpf embryos. Filipin staining of 2 dpf embryos demonstrated reversal of unesterified cholesterol accumulation in the YSL of mutant larvae expressing *npc1*. Scale bar: 200 μm.

Accumulation of unesterified cholesterol was reported in zebrafish embryos injected with *npc1* morpholinos as early as at 12 hours post-fertilization (hpf) (Schwend et al., 2011). Therefore, we examined the accumulation of unesterified cholesterol in *npc1* mutants over the course of embryonic and early larval stages. At 1 dpf, similar filipin-positive staining, outlining cell membranes, was observed in both control and mutant larvae (Fig. 5A). However, by 2 dpf, filipin-positive puncta were observed in the yolk area of *npc1* mutants. These filipin-positive puncta were not observed in controls (Fig. 5A). The accumulation of unesterified cholesterol in the embryonic tissues of *npc1* mutants was first observed at 3 dpf, with most of the filipin staining concentrated along the lateral line (Fig. 5A). After 3 dpf, *npc1* mutants accumulated more unesterified cholesterol. Large unesterified cholesterol accumulations were found throughout the entire trunk area in the *npc1* mutants by 5 and 7 dpf (Fig. 5A). We further evaluated this defect in intracellular cholesterol transport during embryonic development by injecting TopFluor-labeled cholesterol into the yolk of 1-cell-stage embryos. At 7 dpf, we observed accumulation of the TopFluor-positive puncta along the notochord and the ventral edge of the trunk in *npc1^m/m^* larvae (Fig. 5B). These TopFluor-positive puncta were not observed in either *npc1^+/+^* or *npc1^+/m^* larvae. Since the accumulation of unesterified cholesterol was initially observed in the yolk area of *npc1* mutants, it was important to determine whether the accumulation occurred in the yolk syncytial layer (YSL) or in the yolk itself. The YSL is a transient extraembryonic tissue connecting the yolk to the embryonic tissues, serving as a center for early embryonic patterning as well as the nutrient transportation intermediate from the yolk (Carvalho and Heisenberg, 2010). To answer this question, we injected pCS2+ plasmid containing zebrafish full-length *npc1* cDNA into the YSL just after formation at 3.5 hpf. Plasmid DNA injected into the YSL stays exclusively within this structure, thus allowing us to determine the location of the unesterified cholesterol accumulation. At 2 dpf, *npc1* mutants injected with control pCS2+ plasmid expressing *EGFP* cDNA exhibited significant unesterified cholesterol accumulation in the yolk area, as evidenced by filipin staining. In contrast, when *npc1* mutants were injected with a plasmid expressing *npc1* cDNA, the accumulation of unesterified cholesterol in the yolk area was significantly reduced (Fig. 6). Thus, unesterified cholesterol accumulation can be first observed in the YSL. In addition, these experiments clearly demonstrate that the cholesterol accumulation phenotype can be rescued by expression of *npc1*.

### *npc1^m/m^* neuromasts demonstrate increased LysoTracker staining

**Fig. 7.**
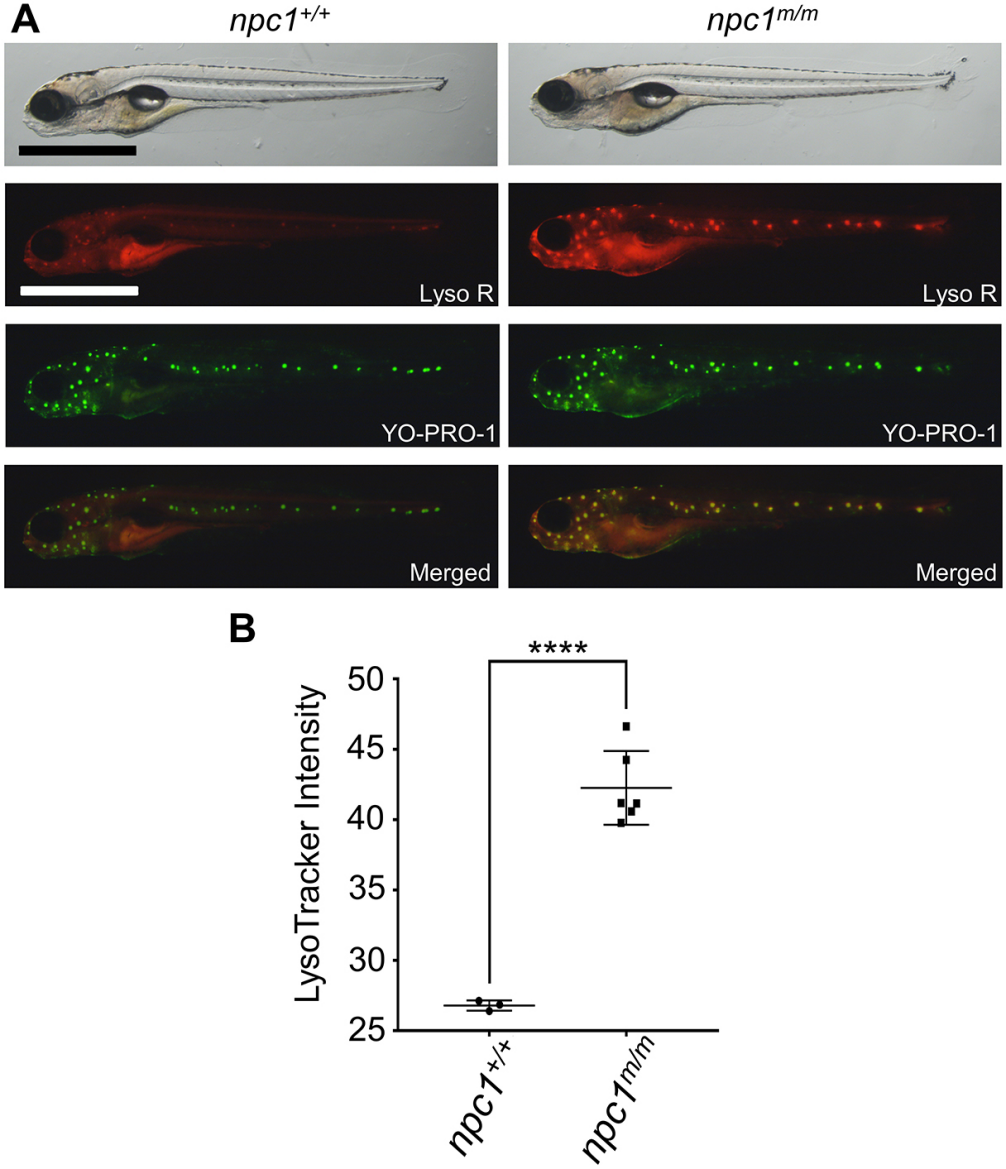
**Intense LysoTracker staining in *npc1^m/m^* lateral line neuromasts.** (A) Vital staining of 5 dpf *npc1^+/+^* and *npc1^m/m^* larvae stained with LysoTracker Red and YO-PRO-1. The merged image localizes the intense LysoTracker Red staining of acidic organelles to the YO-PRO-1-positive neuromasts. Scale bar: 1 mm. (B) Mean LysoTracker intensity of lateral line neuromasts was quantified from multiple individuals. Note that LysoTracker relative intensity of lateral line neuromasts was significantly increased in *npc1^m/m^* larvae at 5 dpf (*npc1^+/+^*: *n*=3; *npc1^m/m^*: *n*=6). ****P<0.0001 by two-tailed *t*-test.

**Fig. 8.**
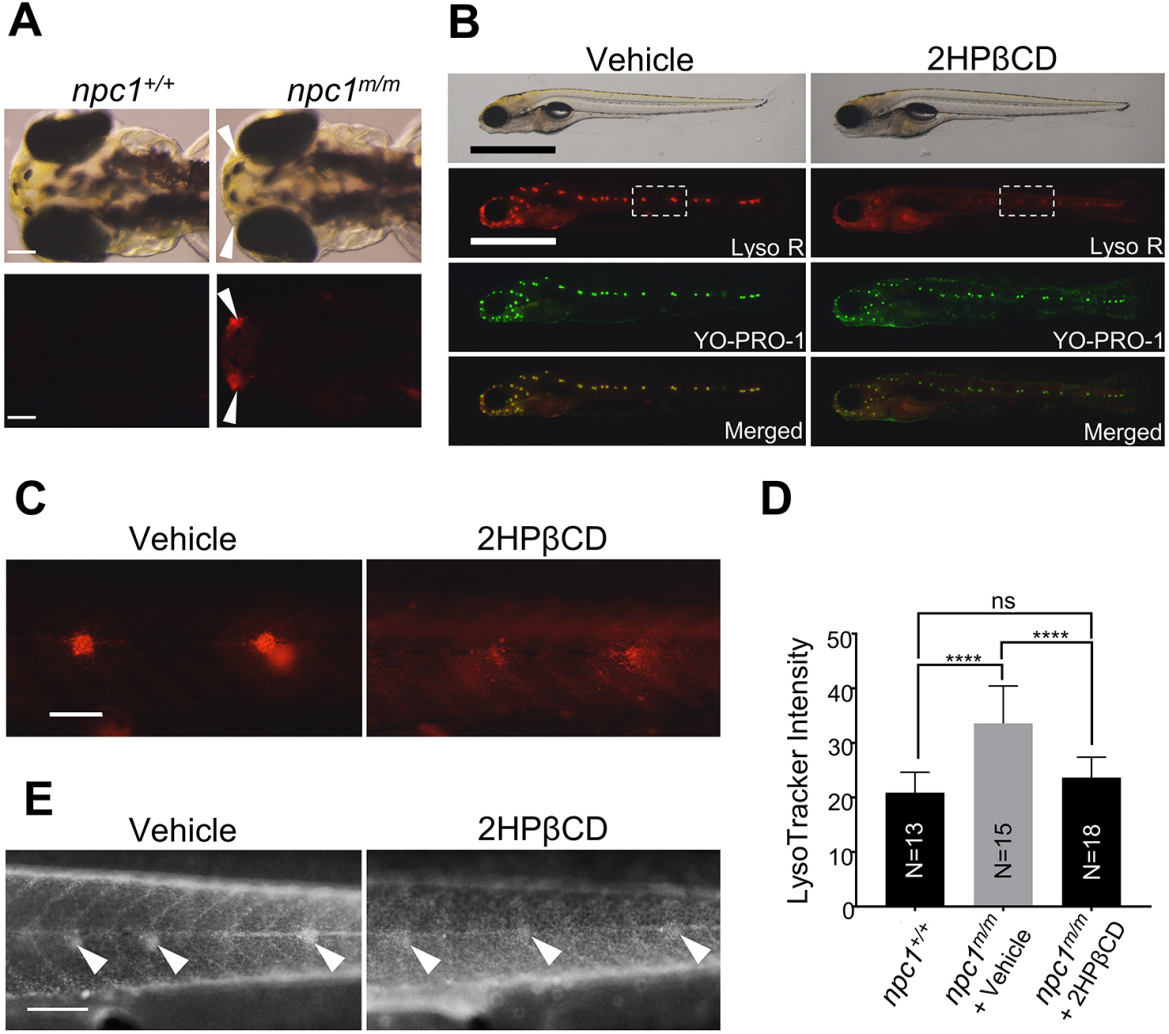
**2HPβCD treatment reduces LysoTracker Red and filipin staining in *npc1^m/m^* neuromasts.** (A) Control and mutant larvae can be differentiated at 3 dpf by intense LysoTracker Red (Lyso R) staining of the olfactory placode (arrowheads) at 3 dpf. Scale bars: 100 μm. (B) Mutant larvae selected by Lyso-R-positive olfactory placode staining were treated for 3 days with vehicle (ddH_2_O) or 2.5 mM 2HPβCD starting at 3 dpf. Neuromast Lyso R staining was markedly decreased in 2HPβCD-treated *npc1^m/m^* larvae. Scale bars: 1 mm. (C) Higher magnification view of Lyso R staining, corresponding to the boxed area in B. Scale bar: 100 μm. (D) Lyso R relative intensity was significantly reduced in *npc1^m/m^* larvae treated with 2.5 mM 2HPβCD at 6 dpf. ****P<0.0001; ns, not significant. Significance was calculated by two-tailed *t*-test. (E) Filipin staining confirmed decreased unesterified cholesterol accumulation in neuromast (arrowheads) of *npc1^m/m^* larvae treated with 2.5 mM 2HPβCD. Scale bar: 200 μm.

It is known that unesterified cholesterol accumulates in the acidic endolysosomal compartment when NPC1 is not functional (Liscum et al., 1989; Wojtanik and Liscum, 2003). This often results in increased size of the endolysosomal compartment as evidenced by increased LysoTracker staining (te Vruchte et al., 2014). LysoTracker Red is a vital, red-fluorescent dye that labels acidic organelles. We visualized the lysosomes in live larval zebrafish using LysoTracker Red staining. At 5 dpf, *npc1* mutants showed a significantly stronger LysoTracker Red signal compared to wild-type larvae, and the areas with the most intense LysoTracker Red signal resembled the distribution of neuromasts (Fig. 7A,B)*.* Neuromasts are the mechanosensory organs found on the skin surface along the lateral line of zebrafish. To confirm that the intense LysoTracker Red staining corresponded to neuromasts, we double-stained larvae with LysoTracker Red and YO-PRO-1. YO-PRO-1 is a vital dye that specifically labels hair cell nuclei in neuromasts (Santos et al., 2006). Although weak LysoTracker Red staining was observed in neuromasts of control larvae, intense LysoTracker Red staining was observed in neuromasts of *npc1* mutant larvae (Fig. 7A). The mean LysoTracker relative intensity of lateral line neuromasts was significantly stronger in *npc1^m/m^* larvae (26.8±0.4) compared with *npc1^+/+^* larvae (42.3±2.6) (Fig. 7B). Next, we examined how early the difference between *npc1* mutants and their siblings could be identified by LysoTracker Red staining. Starting at 3 dpf, live larvae from the intercross of *npc1* heterozygous mutants showed a small increase in LysoTracker Red staining of both the lateral line neuromasts and the olfactory placodes. The small difference in staining made it difficult to easily differentiate control and mutant larvae immediately after staining. However, in contrast to control larvae, LysoTracker Red staining of the mutant larvae olfactory placodes remained strongly positive after a 2-h wash-out period (Fig. 8A). The mutant larvae stained with LysoTracker Red retained the dye for several hours, and their development was not affected by the procedure (data not shown). Thus, LysoTracker Red staining of the olfactory placode followed by a washout period can be used to highly enrich (∼98%) for *npc1^m/m^* larvae.

### LysoTracker Red staining of *npc1^m/m^* larvae provides a robust, rapid, high-capacity *in vivo* screen for potential therapeutic drugs

The ability to identify and select *npc1^m/m^* embryos at 3 dpf combined with an easily recognizable neuromast cell phenotype in viable larvae suggested that the LysoTracker Red staining could be used as a primary readout for drug efficacy in an *in vivo* drug screen. As a proof of principle, we tested the feasibility of using *npc1^m/m^* larvae to screen for potential drugs using 2HPβCD, which has previously been shown to be effective in both mouse and feline models of NPC1 and in a human Phase 1/2 trial (Aqul et al., 2011; Davidson et al., 2009; Liu et al., 2009; Ory et al., 2017; Ramirez et al., 2010; Vite et al., 2015). An enriched population of 3 dpf *npc1^m/m^* larvae was obtained by isolating olfactory placode LysoTracker-Red-positive larvae. These larvae were then treated with either 2.5 mM 2HPβCD or vehicle (ddH_2_O) for 3 days. After 3 days of treatment we double-stained the larvae with LysoTracker Red and YO-PRO-1. At 6 dpf, the LysoTracker Red staining of the neuromast cells was significantly reduced in 2HPβCD-treated mutant larvae compared to vehicle-treated larvae (Fig. 8B,C). The mean LysoTracker intensity of lateral line neuromasts was significantly increased in vehicle-treated mutant larvae (33.6±6.9) when compared with untreated wild-type larvae (20.9±3.7), but it was reduced in 2HPβCD-treated mutant larvae (23.6±3.7) compared with the vehicle-treated mutant larvae (Fig. 8D). Filipin staining confirmed that accumulation of unesterified cholesterol was reduced in 2HPβCD-treated mutant neuromasts (Fig. 8E). Although 2HPβCD treatment caused the reduction of both LysoTracker intensity and filipin staining in neuromasts, it did not alleviate the liver defect as the dark liver phenotype was still observed in either vehicle- or 2HPβCD-treated *npc1* mutant larvae at 7 dpf (Fig. S2). Also, consistent with the lack of tissue penetration by 2HPβCD, the survival rate at 2 weeks was similar in vehicle- and 2HPβCD-treated *npc1^m/m^* zebrafish.

## DISCUSSION

To gain further insight into NPC1 pathology and to obtain an *in vivo* NPC1 model system that would be amenable to high-capacity screening of potential therapeutic compounds, we utilized CRISPR/Cas9-mediated gene targeting to mutate zebrafish *npc1*. Although a morpholino *npc1*-knockdown model has been reported (Louwette et al., 2013; Schwend et al., 2011), to our knowledge, this is the first characterization of a genetic *npc1* zebrafish model. We obtained and characterized 2 independent *npc1* mutant alleles, *npc1^y535^* and *npc1^hg37^*. Both mutations involve small indels that result in a coding frameshift and give rise to a similar phenotype.

Our *npc1* mutant zebrafish model replicates both the early-onset liver disease and the delayed-onset neurological disease seen in human patients. Consistent with what is observed in other model systems, the *npc1* mutant zebrafish show the expected intracellular accumulation of unesterified cholesterol. Intracellular accumulation of unesterified cholesterol was observed as early as 2 dpf in the YSL. The *npc1* mutant larvae appeared normal and were found in expected numbers up to 6 dpf. However, at 7 dpf we noted that the liver tissue appeared dark and liver size was increased in the *npc1* mutant larvae. Histopathological studies showed that the mutant larvae liver tissue was vacuolated and strongly filipin positive, indicating significant intracellular accumulation of unesterified cholesterol. This liver disease likely underlies the significant loss of *npc1* mutant fish immediately after the larval stage. Liver disease has been shown to be associated with the most severe, neonatal form of NPC1, and it is the most common cause of death in neonatal-onset NPC1 patients (Gumus et al., 2017). Many NPC1 patients have a history of prolonged neonatal jaundice and hepatomegaly (Kelly et al., 1993). The neonatal liver disease can lead to cirrhosis or liver failure, or evolve into subclinical chronic liver disease (Kelly et al., 1993). This NPC1 model thus replicates the early-onset liver disease seen in many NPC1 patients.

Although the neonatal NPC1 liver disease can be fatal, the natural history of NPC1 is that the cholestatic liver disease often appears to resolve. Neurological signs/symptoms then manifest later in childhood or adolescence. Neurological manifestations typically include progressive vertical supranuclear gaze palsy, cerebellar ataxia and dementia. Progressive loss of cerebellar Purkinje neurons and axonal spheroids are pathological hallmarks of NPC1 disease (Walkley and Suzuki, 2004). Our *npc1* mutant zebrafish model also manifests a late-onset neurological phenotype. Consistent with the cerebellar ataxia observed in patients, mutant zebrafish showed impaired ability to maintain normal body positioning and discoordinated swimming. The neurologically affected zebrafish die soon after manifesting these signs. Neuropathology at 9 weeks of age demonstrates axonal spheroids and disorganized cerebellar Purkinje neurons in the *npc1* mutant fish. The adolescent/adult zebrafish also have marked growth retardation. This growth failure could be due to either peripheral disease (such as chronic liver disease) or CNS dysfunction impairing feeding.

Previous groups have used morpholinos to knock down *npc1* expression in zebrafish (Louwette et al., 2013; Schwend et al., 2011). Zebrafish embryos injected with *npc1* translation-blocking morpholinos exhibit significant accumulation of unesterified cholesterol in embryonic tissues at 12 hpf (Schwend et al., 2011). These *npc1* morphants manifest delayed epiboly movement and a body axis defect, suggesting the possibility that Npc1 plays important roles on the very first day after fertilization (Schwend et al., 2011). Developmental malformations are not typically observed in human patients. We did not observe these early developmental phenotypes in our genetic NPC1 model. Zebrafish *npc1* is expressed both maternally and zygotically (Schwend et al., 2011). Thus, it is possible that the genetic/morphant phenotypic differences are due to the presence of functional *npc1* maternal transcripts in our genetic model. Owing to failure of the adult *npc1^m/m^* fish to breed, we have not been able to obtain maternal zygotic *npc1* mutants to determine whether they have early developmental defects similar to those reported in the morpholino studies. Alternatively, the observed differences could be due to off-target effects in the morpholino studies or secondary to an acute versus chronic loss of Npc1 function (Boer et al., 2016; Kok et al., 2015; Law and Sargent, 2014).

Zebrafish have been used extensively as *in vivo* models for high-throughput drug screening due to the ability to rapidly assess morphological or behavioral readouts in live animals (Saydmohammed and Tsang, 2018; Wiley et al., 2017). In this paper, we show that the *npc1* mutants displayed increased LysoTracker Red staining of the nasal placode at 3 dpf and increased staining in neuromasts at 6 dpf. These two observations can be combined to provide an efficient, robust, rapid, high-capacity screen. First, we can highly enrich for *npc1* mutant larvae using LysoTracker Red nasal placode staining at 3 dpf. The ability to enrich for mutant larvae prior to drug/compound screening greatly simplifies the use of this model for drug screening by decreasing the need for time-consuming and labor-intensive genotyping. Second, the increased neuromast LysoTracker Red staining observed in mutant larvae provides an easy assay to test the potential *in vivo* efficacy of candidate therapeutic drugs/compounds. The zebrafish lateral line consists of clusters of neuromasts. Neuromasts are a sensory organ that contain mechanosensory hair cells that detect pressure changes due to water flow and nearby objects (Metcalfe et al., 1985; Raible and Kruse, 2000). Neuromast hair cells are functionally and structurally similar to mammalian inner ear hair cells and have been widely studied as a model for ototoxicity (Buck et al., 2012; Santos et al., 2006). The fact that neuromast cells are in direct contact with the surrounding media containing the test compounds is also advantageous with respect to avoiding false negatives due to failure of the drug to penetrate the larvae.

We have demonstrated that LysoTracker Red intensity in neuromasts was significantly reduced in 2HPβCD-treated *npc1* mutants at 6 dpf, suggesting the potential of using this model for high-capacity screening or candidate drug optimization. 2HPβCD is not orally absorbed nor does it cross cellular barriers such as the blood-brain barrier. Thus, we would not anticipate an effect on either the liver or neurological phenotype. A similar compound, methyl-β-cyclodextrin (MβCD), enters cells via fluid-phase endocytosis (Fenyvesi et al., 2014). Thus, the correction of the neuromast LysoTracker Red staining is likely due to proximity of the neuromast cells to the surrounding media. This is a distinct advantage for screening of potential therapeutic compounds that otherwise would have to be delivered systemically.

In this manuscript, we describe the generation of a genetic zebrafish NPC1 model. The NPC1 zebrafish manifest both the early liver disease and later-onset neurological disease characteristic of NPC1. Pathological findings include intracellular accumulation of unesterified cholesterol, vacuolization of hepatocytes, formation of axonal spheroids and apparent disorganization of cerebellar Purkinje neurons. By treating the *npc1* mutant larvae with 2HPβCD, we demonstrated the potential utility and feasibly of using this NPC1 zebrafish model for efficient and rapid *in vivo* screening of candidate therapeutic compounds. This NPC1 zebrafish model adds to and complements other mammalian NPC1 models. It provides a novel tool to both gain insight into pathological processes contributing to NPC1 pathology, and to facilitate the development and translation of potential therapies to NPC1 patients.

## MATERIALS AND METHODS

### Zebrafish husbandry and generation of mutant lines

Zebrafish *npc1* mutants were generated by CRISPR/Cas9-mediated gene targeting. Two targeting regions, located within *npc1* exon 2 (5′-CCGGTCCTGCTGTGCCTCTGCCT-3′) and exon 7 (5′-CCATCAGAGTTTAAGGAGTG-3′), were chosen for sgRNA recognition. Each corresponding sgRNA (Integrated DNA Technologies, Coralville, IA, USA) was injected into wild-type zebrafish embryos at the 1-cell stage together with *cas9* mRNA. Embryos from wild-type EKW and TAB-5 zebrafish were used for targeting exon 2 and exon 7, respectively. For mutation screening, sgRNA-injected F0 embryos were raised to adulthood and outcrossed to either EKW or TAB-5 wild-type adults to obtain F1 embryos. PCR and fragment analysis using genomic DNA from 16 randomly selected F1 embryos were performed to identify potential F0 adults carrying mutations (founders). Additional F1 embryos from the F0 founders were raised to adulthood and screened for mutations by PCR and fragment analysis. Two *npc1* frame-shift mutant alleles were identified during the screening, namely *npc1^y535^* (NM_001243875.1: c.194_201delinsCTGTGCCTC) in exon 2 and *npc1^hg37^* (NM_001243875.1: c933delinsATCAG) in exon 7. All zebrafish lines were maintained in the aquatic animal facility at 28.5°C according to our Animal Use Protocol (AUP), approved by the Institutional Animal Care and Use Committee (IACUC) of the Eunice Kennedy Shriver National Institute of Child and Human Development, MD, USA.

### Genotyping

To genotype *npc1* mutants, genomic DNA was extracted from either whole embryos/larvae or the caudal fin of adults with DNA extraction buffer (10 mM Tris pH 8.2, 10 mM EDTA, 200 mM NaCl, 0.5% SDS and 200 µg/ml proteinase K). Genomic DNA was further diluted 20-fold and then used as the template for genotyping PCR. For the *npc1^y535^* allele, genotyping PCR was carried out using a forward primer npc1Ex2F1 (5′-CCAGCACTGTATCTGGTACGG-3′) and a reverse primer npc1Ex3R1 (5′-ACCAGTCTCGGACACAGCTC-3′). The PCR conditions were: 94°C for 2 min; 35 cycles of 94°C for 30 s, 63°C for 30 s, 72°C for 30 s; and 72°C for 5 min. For the *npc1^hg37^* allele, primers used for genotyping PCR were designed through dCAPS Finder 2.0 online tool (Department of Biology, Washington University, St Louis, MO, USA) to generate an artificial restriction enzyme cutting site on the PCR product. The PCR was carried out using a forward primer Znpc1 Ex7-AvaII-F (5′-TTCTTGACAGCAATCAGCCCCGGTC-3′) and a reverse primer Znpc1 Int7-8-R2 (5′-GAGGGTGTCTGCAGGTTTCACC-3′). The PCR conditions were 94°C for 2 min; 45 cycles of 94°C for 30 s, 63°C for 30 s, 72°C for 30 s; and 72°C for 5 min. PCR products from both *npc1^y535^* and *npc1^hg37^* were digested with restriction enzyme *Ava*II (R0153, New England BioLabs, Ipswich, MA, USA) at 37°C for 8 h. Final digestion products were resolved on 2% agarose gels.

### Live imaging

Live zebrafish larvae or adults were anesthetized by adding 0.16 mg/ml tricaine (A5040, Sigma-Aldrich, St Louis, MO, USA) to the egg or system water prior to imaging. After larvae or adults were anesthetized, they were mounted on a layer on a glass depression slide with 3% methylcellulose solution. Images of live zebrafish were obtained using a Leica MZ16F stereomicroscope (Leica Microsystems, Wetzlar, Germany) with an AxioCam HRc CCD camera (Carl Zeiss, Jena, Germany).

### Oil Red O staining

At 7 dpf, larvae were fixed with 4% paraformaldehyde in 1× PBS at 4°C overnight. After rinsing with 1× PBS 3 times, fixed larvae were incubated sequentially with 85% and 100% propylene glycol (P4347, Sigma-Aldrich) for 10 min at room temperature. Larvae were transferred to ORO staining solution [0.5% ORO (O0625, Sigma-Aldrich) in 100% propylene glycol] and placed on a platform rocker for overnight staining at room temperature. Stained larvae were destained with gradual transition from 100% propylene glycol to 1× PBS and eventually transferred to glycerol for imaging. Images were obtained using a Leica MZ16F stereomicroscope (Leica Microsystems) with an AxioCam HRc CCD camera (Carl Zeiss).

### Histology

Larval (7 dpf) or adult (9-week-old) zebrafish were fixed with 4% paraformaldehyde in 1× PBS for 24 h. After fixation, samples were dehydrated sequentially and eventually stored in 70% ethanol at −20°C. Paraffin embedding and microtome sectioning were performed to obtain tissue sections for H&E staining (Histoserv, Germantown, MD, USA). Stained sections were imaged using an Axioplan 2 compound microscope with an AxioCam 105 Colors CCD camera (Carl Zeiss).

### Whole-mount immunofluorescence and western blot for Npc1

Rabbit monoclonal anti-Npc1 antibody (ab134113, Abcam, Cambridge, UK) was used for both immunofluorescence (1:100) and western blot (1:1000). For immunofluorescence, zebrafish larvae were fixed with 4% paraformaldehyde in 1× PBS at 4°C overnight. Fixed larvae were rinsed extensively with 1× PBT (0.5% Triton X-100 in 1× PBS) and subsequently blocked with blocking solution (2% BSA, 1% DMSO, 0.5% Triton X-100, 0.5% goat serum in 1× PBS) at room temperature for 1 h. Larvae were incubated with the primary antibody diluted in the blocking solution at 4°C overnight. After rinsing the larvae 3 times with 1× PBT, they were incubated with the secondary antibody (goat anti-rabbit IgG–Alexa-Fluor-594) at 4°C overnight. Stained larvae were rinsed 3 times with 1× PBT and 1× PBST (0.1% Tween-20 in 1× PBS). Immunofluorescence images were taken using a Zeiss Observer Z1 inverted compound fluorescence microscope with a Calibri.2 LED lighting system and a CCD camera (Carl Zeiss). For western blot analysis, livers from three 9-week-old adult zebrafish of the same genotype were dissected and pooled together in RIPA buffer for protein extraction. Total protein concentration was determined by BCA assay (23227, Thermo Fisher Scientific, Waltham, MA, USA). SDS-PAGE separation was done by running 40 μg of total protein per well on NuPage 4-12% Bis-Tris Protein Gels (NP0322, Thermo Fisher Scientific). Proteins were then transferred to nitrocellulose paper using an iBlot 2 transfer apparatus (Thermo Fisher Scientific). Blots were stained with Ponceau S for loading control before the incubation with primary antibody at 4°C overnight. Secondary antibody incubation was done by incubating blots with horseradish peroxidase (HRP)-conjugated goat anti-rabbit IgG (1:10,000) at room temperature for 2 h after they were rinsed several times with 1× TBST. For signal detection, Clarity Western ECL Substrate (1705061, Bio-Rad, Hercules, CA, USA) was used to develop luminescence on the blot.

### Filipin staining

Embryos/larvae were fixed with 4% paraformaldehyde in 1× PBS at 4°C overnight. After extensive rinsing with 1× PBS, fixed embryos/larvae were then stained with filipin staining solution containing 0.5 mg/ml filipin (08707, Polysciences, Warrington, PA, USA) and 1% goat serum in 1× PBS for 2.5 h at room temperature. Stained embryos/larvae were rinsed and stored in 1× PBST before imaging. Images were taken using a Zeiss Observer Z1 inverted compound fluorescence microscope with a Calibri.2 LED lighting system and a CCD camera (Carl Zeiss).

### Labeled cholesterol and DNA microinjection

A total of 20 mM TopFluor-cholesterol (810255P, Avanti Polar Lipids, Alabaster, AL, USA) dissolved in DMSO was injected into the yolk of embryos at the 1-cell stage to label the cholesterol distribution in live zebrafish larvae. Each embryo was injected with 20 pmol of TopFluor-cholesterol. As a control, FITC-BSA (A23015, Thermo Fisher Scientific) was injected into the yolk of embryos at the 1-cell stage. Injected embryos were raised to 7 dpf and the live larvae were then imaged using an MZ16F stereomicroscope (Leica Microsystems) and an AxioCam HRc CCD camera (Carl Zeiss). For DNA microinjection, 50 pg of pCS2+-*EGFP* or pCS2+-*npc1* plasmid containing the full-length *EGFP* or *npc1* cDNA driven by a CMV promoter was injected into the YSL of embryos at 3.5 hpf. Injected embryos were collected and fixed at 2 dpf for filipin staining.

### Vital dye staining

LysoTracker Red DND-99 (L7528, Thermo Fisher Scientific) was used to stain lysosomes and other acidic organelles in live zebrafish larvae. Zebrafish larvae at 3-7 dpf were rinsed with fresh egg water twice before bathing in the egg water containing LysoTracker Red DND-99 (1:1000 dilution). Larvae were incubated with the dye for 1 h in the dark. After the staining, larvae were rinsed 3 times with fresh egg water. YO-PRO-1 iodide (Y3603, Thermo Fisher Scientific) was used for labeling neuromast hair cell nuclei (1:500 dilution). Images were obtained using a Leica MZ16F stereomicroscope (Leica Microsystems) equipped with an AxioCam HRc CCD camera (Carl Zeiss). The mean intensity of LysoTracker Red in the lateral line neuromasts was quantified per individual larvae via ImageJ particle analysis with the following constraints: size (inch)^2^=0.001-0.1, circularity=0.11-1.00.

### 2-hydroxypropyl-beta-cyclodextrin treatment

2-hydroxypropyl-beta-cyclodextrin (2HPβCD; C0926, Sigma-Aldrich) was dissolved in ddH_2_O as a 100 mM stock solution. For the treatment, 15-20 zebrafish larvae were placed in 60 mm-diameter glass Petri dishes with the egg water. A 100 mM 2HPβCD stock solution was then diluted to the working concentration for each treatment group. Larvae were incubated in the egg water with 2HPβCD for 3 days at 28.5°C.

### Statistical analyses

All graphs were plotted as mean±standard deviation (s.d.). Genotype distribution of the *npc1^+/m^* intercross was analyzed by chi square. Other differences between experimental groups were analyzed by two-tailed Student’s *t*-test. *P*-values <0.05 were considered statistically significant.

## Supplementary Material

Supplementary information

First Person interview
