# Supplementary material for: Modeling Niemann-Pick disease type C1 in zebrafish: a robust platform for in vivo screening of candidate therapeutic compounds
Source: Dis Model Mech. 2018 Aug 15;11(9):dmm034165. doi: 10.1242/dmm.034165 (PMC6176986; doi:10.1242/dmm.034165)
Supplement: Supplementary information [file dmm-11-034165-s1.pdf]

FIRST PERSON

First person – Wei-Chia Tseng

First Person is a series of interviews with the first authors of a selection of papers published in Disease Models & Mechanisms, helping early-career researchers promote themselves alongside their papers. Wei-Chia Tseng is first author on 'Modeling Niemann-Pick disease type C1 in zebrafish: a robust platform for *in vivo* screening of candidate therapeutic compounds', published in DMM. Wei-Chia is a Postdoctoral Fellow in the lab of Forbes D. Porter at National Institutes of Health, Department of Health and Human Services, Bethesda, USA, using zebrafish models to study lysosomal storage diseases.

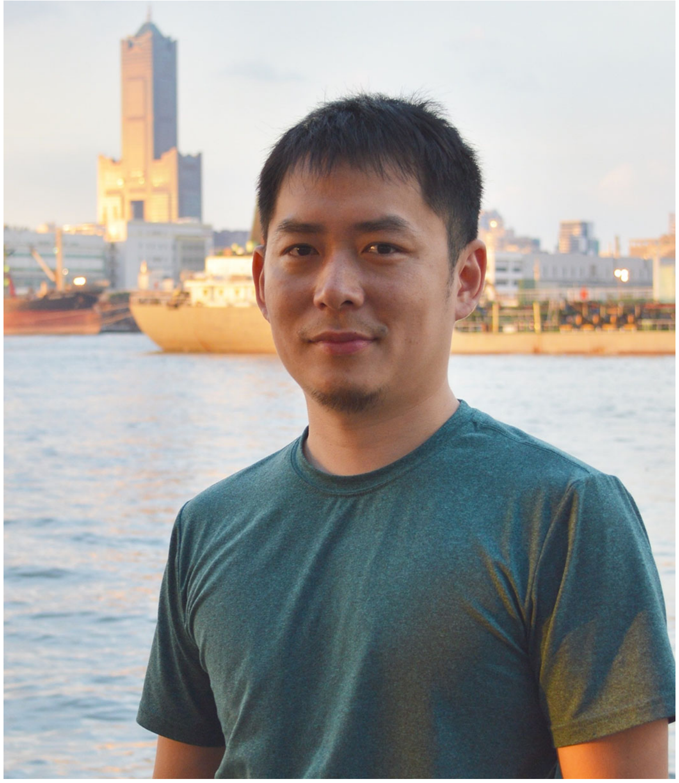

Wei-Chia Tseng

How would you explain the main findings of your paper to non-scientific family and friends?

Niemann–Pick disease, type C1 (NPC1) is a rare, often fatal, disease caused by mutations in the *NPC1* gene, which is essential for transporting cholesterol across vesicles called lysosomes within the cell. Mutations in the *NPC1* gene result in the accumulation of cholesterol and lipids in these lysosomes, and further cause liver defects and neurological defects in the brain. Currently there is no FDA-approved drug available for the treatment of NPC1. Therefore, speeding up the drug-screening process for potential NPC1 treatments is very important. We generated a zebrafish genetic NPC1 model that shows some of the same disease features found in NPC1 patients. We have shown that our zebrafish NPC1 model can be used as a new platform to boost the screening of candidate drugs for treating NPC1.

What are the potential implications of these results for your field of research?

Previously, most of the drug screening and development of potential NPC1 treatments has been performed using an *in vitro* cell culture model, followed by testing a few candidates in mammalian models. However, it takes a long time to conduct drug tests in mammals, and drugs that show efficacy in cell culture may not behave the same way in mammals. Therefore, an intermediate model for testing candidate drugs identified by *in vitro* screening before moving on to mammalian model testing is necessary to reduce the false-positive rate. We have shown that the lateral line neuromasts of our *npc1*-null mutant zebrafish larvae provide a great *in vivo* system for screening candidate drugs for treating NPC1. A proof-of-principle experiment with 2HPβCD treatment and staining live *npc1*-null mutant zebrafish larvae with LysoTracker showed that reduction of LysoTracker intensity can be easily captured by fluorescence microscopy, and serves as a readout for drug treatment. The results suggest that our zebrafish NPC1 model can be used as an *in vivo* model to bridge the gap between *in vitro* and mammalian models for NPC1 drug screening.

Wei-Chia Tseng's contact details: Section on Molecular Dysmorphology, Division of Translational Research, Eunice Kennedy Shriver National Institute of Child Health and Human Development, National Institutes of Health, Department of Health and Human Services, Bethesda, MD 20892, USA.
E-mail: wei-chia.tseng@nih.gov

"The advantages of using zebrafish as the model system include larger clutch size and the transparency of embryos and larvae."

What are the main advantages and drawbacks of the model system you have used as it relates to the disease you are investigating?

The advantages of using zebrafish as the model system include larger clutch size and the transparency of embryos and larvae. We can easily obtain 100 *npc1* homozygous mutants for drug screening in a single clutch from the intercross of *npc1* heterozygous mutants, while only getting one or two *npc1* mutants per litter for mice. The transparency of embryos and larvae allows us to examine the phenotype of internal structures in zebrafish *npc1*-null mutant larvae when they are still alive. Moreover, we took the advantage of neuromasts, which are unique mechanosensory organs located on the skin of zebrafish but not in mammals, as the target for the drug treatment. The accessibility of neuromasts to the external environment makes it easier for drugs to be delivered into cells. Analyzing the effect of drug treatment is also easier due to the high concentration of LysoTracker-positive spots in *npc1* mutant neuromasts. The drawback of using zebrafish is the generation time of about two to four months, making it even slightly slower than the generation time of mice.

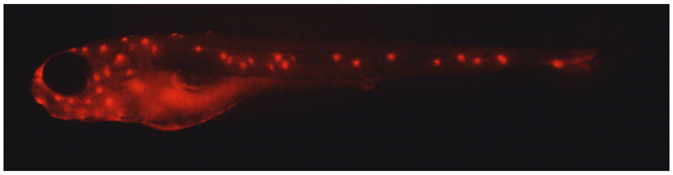

A live zebrafish *npc1* mutant larva at 5 days post-fertilization, stained with LysoTracker Red, showing intense staining in neuromasts.

What has surprised you the most while conducting your research?

Our *npc1* mutants exhibited very intense LysoTracker staining in neuromasts as well as in olfactory placodes. This phenotype is unique and useful for sorting the live *npc1* mutant larvae from their wild-type and heterozygous mutant siblings.

Describe what you think is the most significant challenge impacting your research at this time and how will this be addressed over the next 10 years?

The most significant challenge of studying Niemann–Pick disease, type C1 is that the disease varies widely in both symptoms and the age at onset between individuals. Some patients develop severe, often fatal, visceral manifestations such as the liver defect very early in their life, while other patients develop variable neurological defects at ages ranging from early childhood to adulthood. The cause of the variability is still not clear. Over the next three to five years, we will understand more about the mechanism of disease progression as new pathways or proteins involved in the process are identified. This will help us to develop and test potential therapeutic methods for a variety of NPC1 patients over the next decade.

What's next for you?

I will continue to work on screening candidate drugs for treating NPC1 using our zebrafish model. At the same time, exploring the pathological mechanism of the disease in the zebrafish model is also important as it will allow me to find potential targets for therapeutic intervention. My goal is to identify drugs that reduce NPC1 pathological features significantly in our zebrafish model and test those drugs in mammalian NPC1 models.

Reference

Tseng, W.-C., Loeb, H. E., Pei, W., Tsai-Morris, C.-H., Xu, L., Cluzeau, C. V., Wassif, C. A., Feldman, B., Burgess, S. M., Pavan, W. J. and Porter, F. D. (2018). Modeling Niemann-Pick disease type C1 in zebrafish: a robust platform for *in vivo* screening of candidate therapeutic compounds. *Dis. Model. Mech.* **11**, dmm034165.
